# Rare Isolated Renal Involvement with Marginal Zone B Cell Lymphoma: A Case Report with Literature Review of Contemporary Management Strategies

**DOI:** 10.7759/cureus.3560

**Published:** 2018-11-08

**Authors:** Muhammad J Khalil, Mustafa N Malik, Maryam Ahmed, Abdul Rafae, Faiz Anwer

**Affiliations:** 1 Hematology and Oncology, The University of Arizona, Tucson, USA

**Keywords:** marginal zone b-cell lymphoma, chemotherapy, renal insufficiency

## Abstract

Marginal zone B cell lymphomas are divided into nodal, extranodal and splenic types. Renal involvement by extranodal B cell lymphoma is extremely rare with an incidence of about 0.1%. We present a case of a 79-year-old Caucasian male with progressive renal failure and isolated left renal extranodal marginal zone lymphoma. Asymptomatic immunoglobulin (Ig) M monoclonal gammopathy along with bone marrow involvement by lymphoma was observed. Contemporary management options including radiotherapy (RT), chemotherapy, immune-modulating agents and novel chemotherapy-free regimens.

## Introduction

Marginal zone lymphomas (MZL) are low-grade non-Hodgkin B cell lymphomas (NHL) classified into three subcategories such as extranodal marginal zone lymphoma (EMZL), also called mucosal-associated lymphoid tissue (MALT) lymphoma, nodal marginal zone lymphoma (NMZL) and splenic marginal zone lymphoma (SMZL). EMZLs account for 8% of all NHL cases [1]. Most commonly, the EMZLs are found in the stomach, followed by spleen, eye, adnexa, lungs, skin, salivary glands, thyroid, small intestine, breast, synovium, dura and soft tissues. As kidneys lack the lymphoid tissues, renal involvement with EMZLs is extremely rare. Only a handful of such cases have been reported [2]. B cell lymphomas also present with paraproteinemia [3]. We report a case of a Caucasian male who presented with a progressive decline in renal function associated with isolated left renal involvement with marginal zone B cell lymphoma and asymptomatic immunoglobulin (Ig) M monoclonal gammopathy. Moreover, we reviewed the contemporary management options for MZLs.

## Case presentation

**Figure 1:**
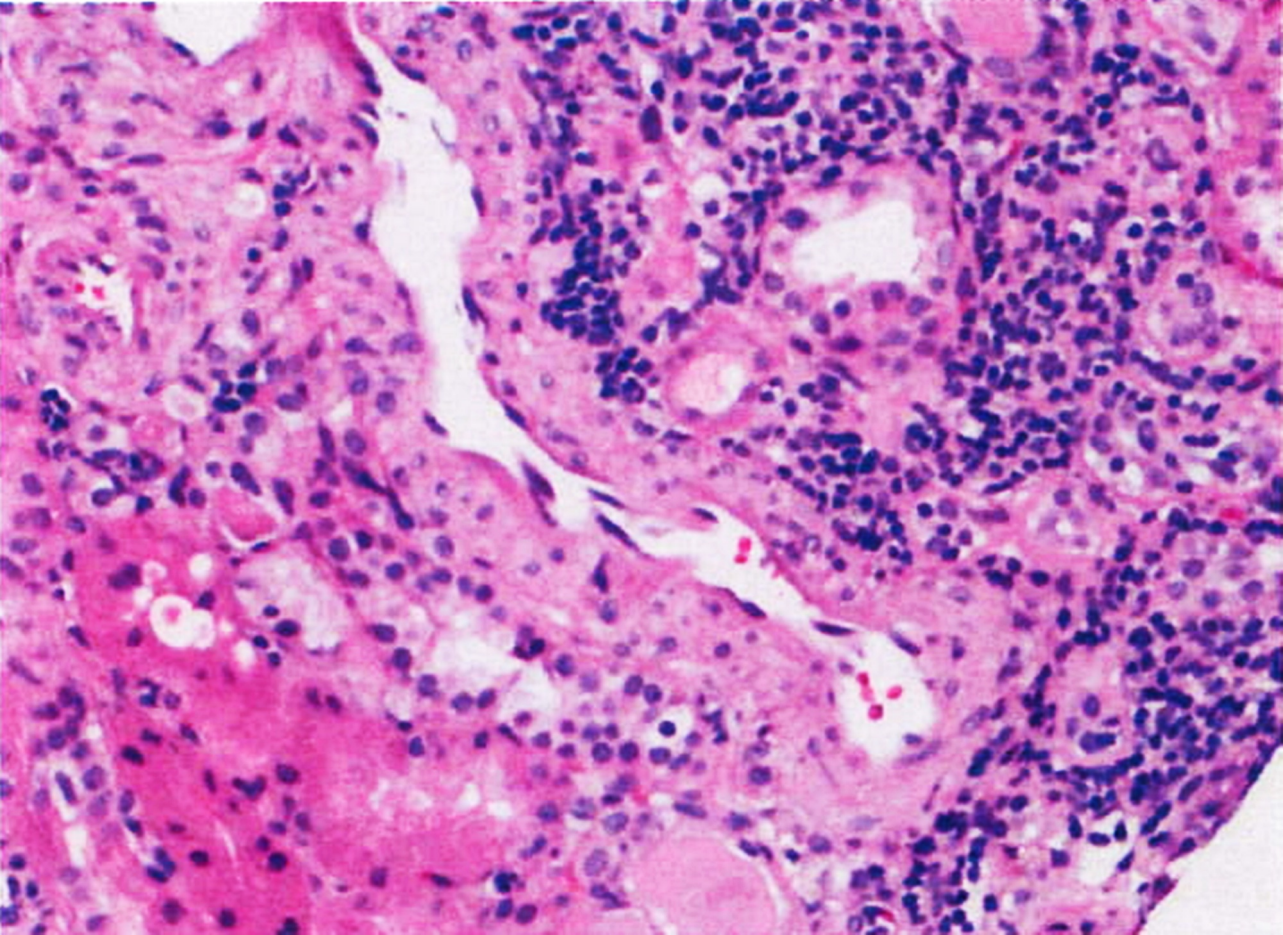
Interstitial inflammation with lymphocytes

A 79-year-old asymptomatic Caucasian male presented with progressive renal failure with abnormal serum creatinine (range of 1.82 to 2.18) in July 2012. Complete blood count (CBC) showed red blood cell count (RBC) of 3.36 x 108/uL, hemoglobin 10.9 g/dl, hematocrit 33.1%, mean corpuscular volume (MCV) 99 fL, mean corpuscular hemoglobin (MCH) 32.8 pg, mean corpuscular hemoglobin concentration (MCHC) 33.0%, red cell distribution width (RDW) 15.8% and white blood cell count (WBC) 4,000/uL, with differentials: neutrophils 50%, lymphocytes 37%, monocytes 8%, eosinophils 4%, basophils 1% and platelet count of 178,000/uL. Comprehensive metabolic profile (CMP) revealed: glucose 96 mg/dL, blood urea nitrogen 23 mg/dL, creatinine 2.18 mg/dL, sodium 140 mmol/L, potassium 4 mmol/L, chloride 107 mmol/L, carbon dioxide 25 mmol/L, albumin 4.4 g/dL, calcium 9.1 mg/dL, bilirubin (total) 0.8 mg/dL, phosphorus 3.3 mg/dL and magnesium 2.7 mg/dL. Computed tomography (CT)-guided needle biopsy of the left kidney showed severe lymphocytic inflammation mainly in the areas of tubular atrophy and interstitial fibrosis (Figure 1).

**Figure 2:**
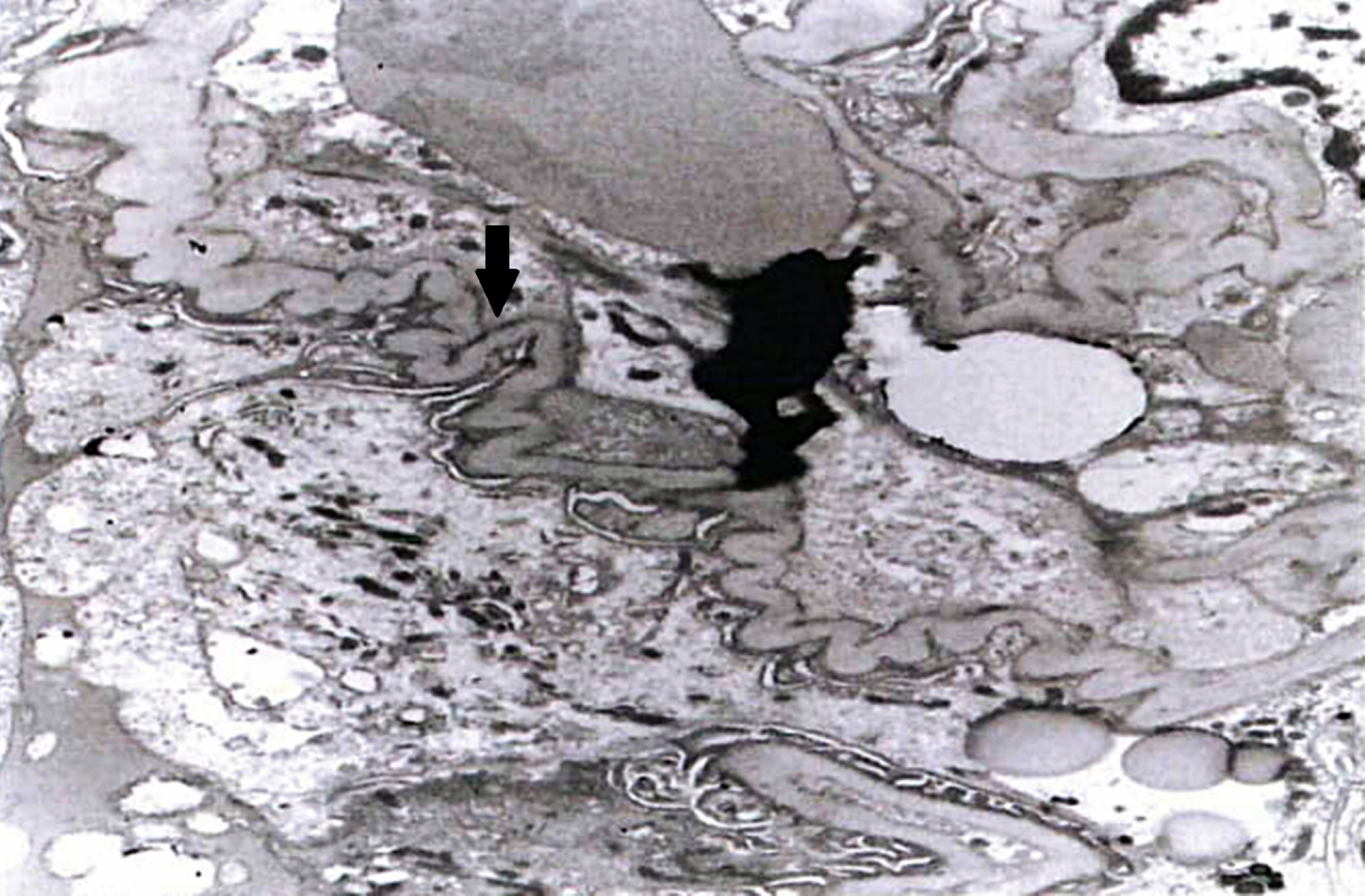
Wrinkling and folding of glomerular capillary loops

Immunohistochemical staining showed atypical lymphocytes that were positive for CD20 and negative for CD5, CD10, CD19, CD22 and CD23. Molecular testing revealed an Ig heavy chain gene rearrangement; these findings were consistent with marginal zone B cell lymphoma. Furthermore, immunofluorescence (IF) microscopy demonstrated seven glomeruli with diffuse pseudo-linear staining of the glomerular capillary loops for albumin (1+). Glomerular staining for IgG, IgA, IgM, C3, C1q and kappa or lambda light chain was negative. Interstitium stained positive for fibrinogen, while protein casts were stained positive for IgA (3+), kappa light chain (3+) and lambda light chain (3+). By electron microscopy (EM), the glomerular basement membrane had a normal trilaminar structure, the mean thickness was within the normal range without electron-dense deposits or tubuloreticular inclusions and the majority of podocytes foot processes were intact (Figure 2). No immune complexes were detected by IF or EM.

**Figure 3:**
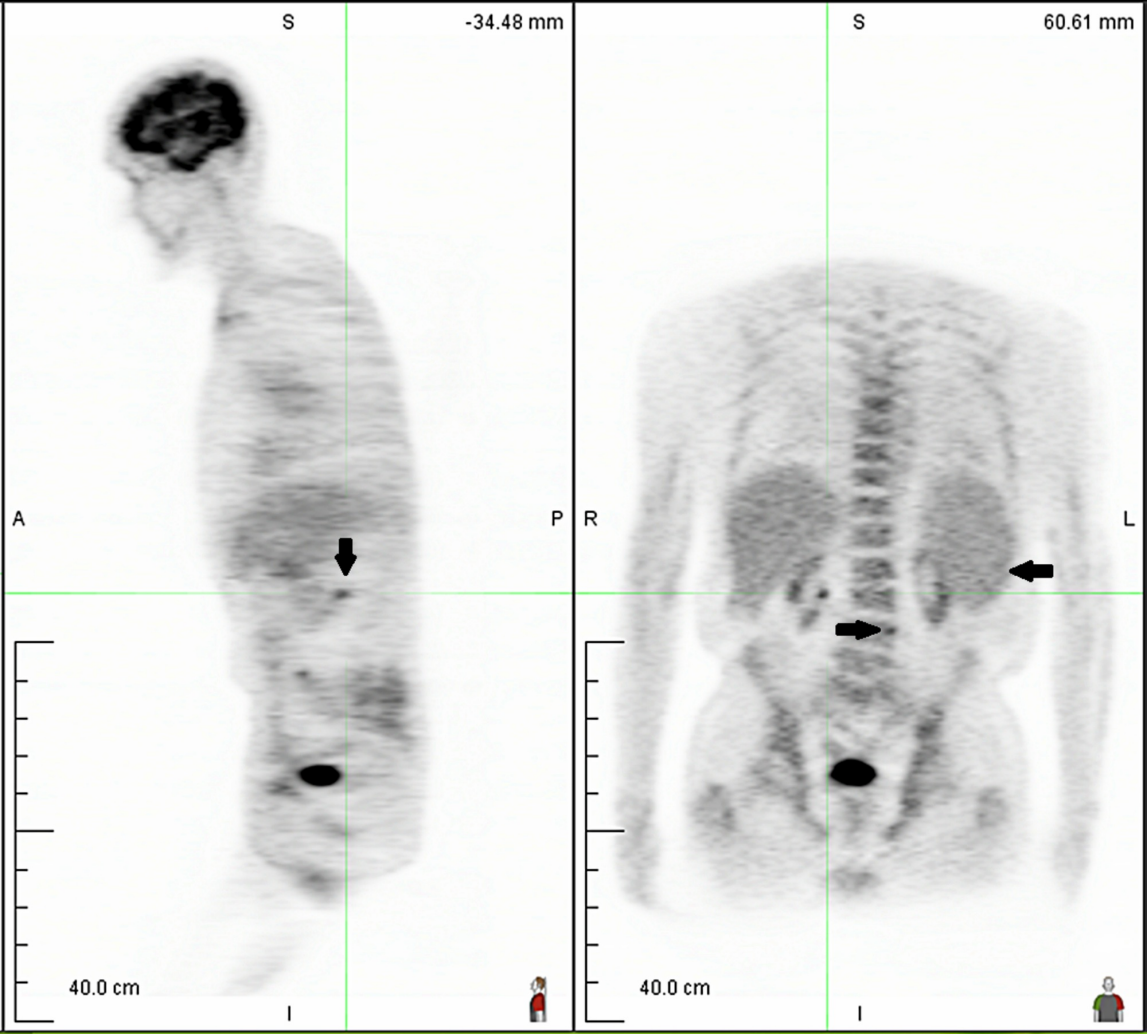
PET scan showing mild enlargement of retroperitoneal lymph nodes PET: positron emission tomography

On serum protein electrophoresis (SPEP) and immunofixation, we found IgM kappa monoclonal gammopathy and serum M-protein level of 0.3 g/dL. Quantitative immunoglobulin testing was performed for IgG (699 mg/dL), IgA (51 mg/dL) and IgM (470 mg/dL). Serum free light chain (SFLC) showed kappa (37.9 mg/L), lambda (1.58 mg/L) and kappa/lambda ratio of 23.99. Urine protein electrophoresis (UPEP) showed a protein level of 91 mg/dL and M-spike of 18.1%. Bone marrow (BM) biopsy showed 30% BM involvement by clonal B cells, morphologically consistent with marginal zone B cell lymphoma. Positron emission tomography (PET) scan was found normal in 2012 and 2013. The patient was treated with five doses of weekly rituximab and one dose of rituximab-bendamustine combination, but renal function worsened and treatment was stopped in 2013. The patient was monitored closely without further treatment until 2017. PET scan (2017) showed a mild enlargement of the retroperitoneal lymph nodes (aortocaval from 1 x 0.8 to 1.4 x 1.2 cm, left periaortic from 1.4 x 0.5 to 1.3 x 1.0 cm) and a partially calcified subcarinal node from 3.1 x 1.1 to 3.5 x 1.5 cm (non-avid), and the spleen size increased from 12.2 to 14.2 cm in cubic centimeter dimension (Figure 3). One dose of vincristine and prednisone was given at that time, but renal function declined further. The patient was offered bortezomib/dexamethasone or ibrutinib for further treatment consideration, which he declined.

## Discussion

Renal infiltration by lymphoma is rare, and such cases are often clinically silent [2]. EMZLs are more common than NMZLs or SMZLs and are subdivided into gastric and non-gastric types. The gastric EMZLs are caused by sustained inflammation with bacterial infection (*Helicobacter pylori)*. Etiologies for non-gastric EMZLs include *Borrelia burgdorferi* infection in cutaneous EMZLs, *Chlamydophila psittaci* infection in ocular EMZLs and *Campylobacter jejuni* infection in small intestinal EMZLs. A higher prevalence of hepatitis C virus (HCV) infection has also been reported in patients with MZLs, particularly of the splenic and nodal types, suggesting a possible causative role of the HCV in MZLs [1].

As no lymphatic tissues are present in the renal parenchyma, the theories about the renal involvement with lymphoma suggest that either the lymphocytes migrate from the lymphatics in the renal capsule or the presence of chronic inflammation provides a platform for long-standing antigenic stimulation, thereby attracting the lymphoid cells and developing into a lymphoma. Mostly, the MZLs present as Ann-Arbor stage IE disease, and BM and peripheral lymph node involvement are rather uncommon. BM involvement has been reported in up to 20% of cases of MZLs [3].

EMZLs are indolent and have a tendency to remain localized to the site of origin for an extended period of time. It has the potential for a systematic spread and can transform to an aggressive B cell lymphoma. Histological transformation to large B cell lymphoma has been reported in approximately 10% of the cases [3]. Patients with EMZL have a relatively good prognosis with a median survival >10 years. Our patient had an indolent clinical behavior of MZL but was diagnosed with isolated left renal involvement at the time of diagnosis.

Treatment options depend on the site of organ involvement. For gastric EMZLs, options include *H. pylori* eradication therapy, radiotherapy (RT), chemotherapy and immune-modulating agents. For early-stage *H. pylori-*positive EMZL, *H. pylori* eradication therapy is recommended, followed by endoscopic surveillance. For advanced-stage *H. pylori*-positive gastric EMZL, treatment with *H. pylori *eradication therapy is recommended, followed by observation until the development of symptoms at which time chemotherapy is initiated. Initial treatment with local RT is recommended in patients with early-stage *H. pylori*-negative gastric EMZL.

Non-gastric EMZLs treatment depends on the organ involved and the stage of the disease. Limited-stage EMZL is generally treated with locoregional RT or managed expectantly. Surgery is typically used for diagnostic purposes only but may play a role in the treatment of tumors in areas not conducive to RT (e.g. localized lesion in the lung). Single-agent rituximab is an alternative for the treatment of sites that cannot be treated with RT. Advanced-stage diseases are treated with rituximab or combination chemotherapy. Chemotherapy or chemoimmunotherapy is given to newly diagnosed patients who show no response to single-agent rituximab or have disease relapse.

Rituximab, in combination with bendamustine, demonstrated an overall response rate (ORR) of 84%, with a six-year progression-free survival (PFS) of 54% and an overall survival (OS) of 72% for newly diagnosed cases. For relapsed/ refractory MZL, bendamustine as a single agent was effective with an ORR of 76%, and the median duration of response was 10 months [4]. Other chemotherapy combinations include rituximab/cyclophosphamide/vincristine/ prednisone (R-CVP), rituximab/fludarabine (FR) and fludarabine/cyclophosphamide/rituximab (FCR). These regimens have shown excellent efficacy with an ORR of 88%, 83% and 78.3%, respectively [5-6].

Obinutuzumab, an anti-CD20 antibody, acts by inducing direct cell death and showed enhanced antibody-dependent cell-mediated cytotoxicity (ADCC). In combination with bendamustine, it has shown a statistically significant (*p* = 0.0001; 95% confidence interval (CI) = 0.40-0.74) increase in PFS over single-agent bendamustine (22.5 months vs. 14.9 months at two years) in 396 patients with indolent lymphomas that were resistant to rituximab (hazard ratio = 0.55) [7].

Ibrutinib, a Bruton’s tyrosine kinase inhibitor, is the first oral therapy approved by the Food and Drug Administration (FDA) for the management of relapsed/refractory (R/R) marginal zone lymphoma. It acts by inhibition of the B cell receptor (BCR) signaling pathway, an important pathway responsible for lymphoma genesis. A phase II study included 63 patients having R/R MZLs treated with ibrutinib. About 50% of the patients had EMZL, and about a quarter patients had SMZL. Ibrutinib has shown excellent efficacy in these patients with an ORR of 48% and median PFS of 14.2 months [8].

Bortezomib, a proteasome inhibitor, has shown a significant single-agent activity in R/R MZLs with ORR of 48% [9]. In a randomized clinical trial involving 81 patients with R/R MZL, bortezomib in combination with rituximab was administered. The patients were randomly assigned into two groups with bortezomib (1.3 mg/m^2^) twice weekly in group A and bortezomib (1.6 mg/m^2^) once weekly in group B. Both groups also received rituximab (375 mg/m^2^) weekly for 4 weeks. Group A showed an ORR of 49%, while group B showed an ORR of 43% [10].

Rituximab, when used in combination with lenalidomide, has shown excellent efficacy with an ORR up to 80% [11]. Other novel agents with activity against MZLs include high-dose cytarabine (HiDAC) inhibitors, phosphoinositide 3 kinase (PI3K) inhibitors and mammalian target of rapamycin (mTOR) inhibitors. These agents are currently in various phases of clinical trials (Table 1) [12-19]. One clinical trial involving 81 patients with relapsed B cell malignancies including MZL, wherein a novel chemotherapy-free triple regimen including umbralisib (PI3K Δ inhibitor), ibrutinib (Bruton’s tyrosine kinase inhibitor) and ublituximab (glycoengineered anti-CD20 antibody) was used, showed an ORR of 86% [20].

**Table 1:** Drugs in various phases of development in clinical trials for the treatment of non-Hodgkin B cell lymphoma bcl: B cell lymphoma; IV: intravenous; N.S: not specified; PO: per oral; R/R: relapsed/refractory

Agent	Mode of Action	Disease	Phase	Patients	Dose (mg)	Route	Ref
Venetoclax	Proto-oncogene protein c-bcl-2-inhibitors	R/R	II	138	50-800	PO	[14]
Copanlisib	Phosphatidylinositol 3 kinase alpha inhibitors	R/R	II	56	60	IV	[12]
Buparlisib	Phosphatidylinositol 3 kinase inhibitors	R/R	I	18	N.S	PO	[15]
Duvelisib	Phosphatidylinositol 3 kinase delta inhibitors	R/R	III	600	200	PO	[16]
Vorinostat	Histone deacetylase inhibitors	R/R	II	37	N.S	PO	[19]
Idelalisib	Phosphatidylinositol 3 kinase delta inhibitors	R/R	II	125	150	PO	[13]
Lenalidomide	Immunomodulator	R/R	II	56	15-20	PO	[17]
Umbralisib	Phosphatidylinositol 3 kinase delta inhibitors	R/R	II	40	N.S	PO	[18]

Ibrutinib is being investigated in combination with lenalidomide +/- rituximab (clinicaltrials.gov, NCT02532257 and NCT01955499), selinexor (KPT-330), a selective inhibitor of nuclear export (clinicaltrials.gov, NCT02303392), and pembrolizumab, a checkpoint inhibitor (clinicaltrials.gov, NCT02332980) in R/R MZL and other B cell malignancies.

## Conclusions

We report a case of marginal zone B cell lymphoma with isolated left renal involvement in a patient with progressive renal failure and asymptomatic IgM monoclonal gammopathy. MZLs are currently being treated with RT, chemotherapy and chemoimmunotherapy. Recently, chemotherapy-free regimens and other novel agents have gained importance. These agents are currently under development in various phases of clinical trials and may play an important role in the future management of MZLs and other B cell malignancies.
